# Decision-Making Algorithm with Geographic Mobility for Cognitive Radio

**DOI:** 10.3390/s24051540

**Published:** 2024-02-28

**Authors:** Gabriel B. Cervantes-Junco, Enrique Rodriguez-Colina, Leonardo Palacios-Luengas, Michael Pascoe-Chalke, Pedro Lara-Velázquez, Ricardo Marcelín-Jiménez

**Affiliations:** Department of Electrical Engineering, Autonomous Metropolitan University, Iztapalapa, Mexico City 09310, Mexico; brakeencj@gmail.com (G.B.C.-J.); erod@xanum.uam.mx (E.R.-C.); lpalacios@izt.uam.mx (L.P.-L.); plara@xanum.uam.mx (P.L.-V.); rmarcelin@izt.uam.mx (R.M.-J.)

**Keywords:** cognitive radio, decision-making, geographic mobility in cognitive radio, location, handoff management

## Abstract

The proposed novel algorithm named decision-making algorithm with geographic mobility (DMAGM) includes detailed analysis of decision-making for cognitive radio (CR) that considers a multivariable algorithm with geographic mobility (GM). Scarce research work considers the analysis of GM in depth, even though it plays a crucial role to improve communication performance. The DMAGM considerably reduces latency in order to accurately determine the best communication channels and includes GM analysis, which is not addressed in other algorithms found in the literature. The DMAGM was evaluated and validated by simulating a cognitive radio network that comprises a base station (BS), primary users (PUs), and CRs considering random arrivals and disappearance of mobile devices. The proposed algorithm exhibits better performance, through the reduction in latency and computational complexity, than other algorithms used for comparison using 200 channel tests per simulation. The DMAGM significantly reduces the decision-making process from 12.77% to 94.27% compared with ATDDiM, FAHP, AHP, and Dijkstra algorithms in terms of latency reduction. An improved version of the DMAGM is also proposed where feedback of the output is incorporated. This version is named feedback-decision-making algorithm with geographic mobility (FDMAGM), and it shows that a feedback system has the advantage of being able to continually adjust and adapt based on the feedback received. In addition, the feedback version helps to identify and correct problems, which can be beneficial in situations where the quality of communication is critical. Despite the fact that the FDMAGM may take longer than the DMAGM to calculate the best communication channel, constant feedback improves efficiency and effectiveness over time. Both the DMAGM and the FDMAGM improve performance in practical scenarios, the former in terms of latency and the latter in terms of accuracy and stability.

## 1. Introduction

Geographic mobility (GM) models describe the movement of nodes in a specific region during a given time, including changes in speed, direction, and acceleration. GM can thus adapt to a solution’s specific needs, providing improved performance and flexibility. The use of wireless networks comprises two important tasks: location and handoff management. Location management ensures that the location of network nodes can be tracked, while handoff management is responsible for maintaining connections while a node moves from one network to another [1]. This GM management needs to be considered for cognitive radio networks (CRNs), given that the available radio spectrum can considerably change with location and handoff. Consequently, user GM represents a major challenge in CRNs [2] since it can significantly affect performance by interrupting the services, thus reducing service quality.

According to the current state of the art, existing mobility models can be divided into four groups: random, group, route-planned, and time-dependent models [3]. Random mobility models in wireless networks consider the random distribution of nodes within a previously defined simulation area. Each node remains in that location for a randomly selected time within a specified interval [4,5]. Similarly, the group mobility model considers that a group of nodes in a network revolve together around a common point [6,7]. Route-planned models seek to avoid unforeseen changes in speed and direction. The planning of the movements is defined by mathematical equations, in which the nodes are forced to follow these movement patterns [8]. Similarly, time-dependent mobility models are based on mathematical equations in which the nodes depend on an initial time with respect to a previous time, thus avoiding sudden changes in speed and direction [9]. Different prediction schemes can be used to control GM. For an implementation in CRNs, it is a question of finding the best GM prediction technique in order to select the most stable communication path, thus improving general performance and performance reliability.

As a consequence of the relevance of GM for a better performance of the CRN, the proposed novel algorithm, named decision-making algorithm with geographic mobility (DMAGM), includes a detailed analysis of decision-making that considers and involves GM.

In addition, an improved version of the DMAGM is also proposed where feedback of the output is incorporated. This version is named feedback-decision-making algorithm with geographic mobility (FDMAGM). This algorithm shows that a feedback system has the advantage of being able to continually adjust and adapt based on the feedback received. Furthermore, the feedback version helps to identify and correct problems, which can be beneficial in situations where the quality of communication is critical.

Grounded in this research’s particular focus on decision-making, different decision-making mechanisms that include GM were studied making the following contributions:

- Proposing a detailed analysis of decision-making that considers geographic mobility (GM), a parameter that most CRN proposals have not yet explored in depth
- Developing a robust process that reduces latency to find a better communication channel; and
- Providing a feedback function that increases the precision in the selection of a better communication backup channel, based on historical data regarding network behavior through a feedback process that considers information from the evaluations of previously used channels. The value assigned to each channel thus corresponds to a relationship between current information and previous evaluations.

The evaluation process of the proposed algorithm is based on determining the attributes characterizing a communication channel through the Delphi method [2]. The decision-making attributes were proposed based on the criteria reported in the CR literature and compared with [2]. Such criteria include the signal-to-interference plus noise ratio (SINR), the bandwidth (BW), the channel availability probability (AP), the estimated channel time availability (ETA), and the random way-point mobility model (RWPM). These criteria were evaluated for two types of services, i.e., real time (RT) and best effort (BE). Test execution was conducted with an NS-3 simulator, and, in order to contrast the DMAGM and the FDMAGM, some comparisons were made with the algorithms Dijkstra [10], analytic hierarchy process (AHP) [11], fuzzy analytic hierarchy process (FAHP) [12], and modified Dijkstra decision-making algorithm (ATDDiM) [13], in which the results indicate a considerable reduction in the processing time of the proposed algorithms. In addition, there was greater precision in the selection of a communication channel since the feedback process proposed in the FDMAGM contained information regarding the evaluations of the current and previous channels. According to the analysis of the different mobility models that can be considered with the interaction among CRs and PUs, it was possible to determine which mobility model would be appropriate for this scenario. This is based on sets of given data that can be compared with wireless networks in operations such as a cellular network. The characteristics that were considered to determine user movements are speed, acceleration, pause time, and time in a location.

The rest of this paper is organized in the following sections. Section 2 describes the related works. Section 3 describes the proposed decision-making algorithm. Section 4 shows the conducted tests that compare latency with similar algorithms and mobility tests. Lastly, Section 5 presents the conclusions. At the end of this document, a table with the list of acronyms used throughout this paper is shown.

## 2. Related Work

To the best of the authors’ knowledge, none of the studies propose research related to spectrum handoff in CRNs and solving problems associated with geographic mobility (GM). Trigui et al. [14] propose a multi-agent system (MAS) with a mobility management scheme for CRNs that considers user mobility and radio resources in order to provide optimal access and make resource allocation decisions. Obaid et al. [15] propose a cluster-based MAC protocol for CR wireless sensor networks with radio frequency (RF) energy harvesting. The protocol is based on the mobility for spectrum access decision-making. The proposed protocol requires a localization system to determine the users’ current location. Priya and Kannan [16] present a frequency-band selection routing protocol with enhanced spectrum aggregation for cognitive radio ad hoc networks. The protocol considers user mobility, spectrum availability, and quality of service to make optimal routing decisions. Zhao et al. [17] propose a new approach to spectrum management in cognitive radio networks based on predicting spectrum availability to make more efficient spectrum allocation decisions. Hanif et al. [18] propose a new approach to spectrum handoff management in CRNs. This approach is based on analyzing the user traffic pattern to make more efficient spectrum handoff decisions and requires the implementation of a traffic analysis system, a spectrum handoff system, and a coordination system. The study by Jaffar et al. [19] proposes a new approach to spectrum management in heterogeneous CRNs based on user location to make proactive channel selection decisions.

The work proposed by Omer et al. [20] introduces a new approach to channel assignment in CRNs for scalable video streaming. This approach is based on adaptively assigning channels to users based on their bandwidth and quality-of-service requirements. On the other hand, Tlouyamma and Velempini [21] propose a channel selection algorithm (CSA) optimized for improved performance in CRNs. This algorithm considers multiple factors, including channel availability, interference, channel quality, user distance, and channel usage history. Thakur et al. [22] consider a new approach to spectrum mobility in CRNs using spectrum prediction and monitoring techniques. This approach is based on predicting the occurrence of primary users (PUs) and continuously monitoring the spectrum in order to detect the presence of PUs. Meanwhile, Yawada and Dong [23] show an approach to spectrum handoff/mobility (SHMA) in CRNs based on an intelligent decision-making process that takes into account multiple factors, including channel availability, interface, channel quality, user distance, and channel usage history.

Li et al. [24] propose a method based on deep learning for the prediction of user mobility using a preferential exploration and return model as a deep-learning technique to predict the future locations of the nodes. According to the proposal made by Iftikhar et al. [25], a decision-making algorithm using game theory is presented to model spectral mobility, which serves as a switching game that considers whether to change or remain in the channel. Alozie et al.’s scheme [26] presents a strategy to minimize the delay that occurs during spectrum handoff from a backup channel selection mechanism based on fuzzy data. Basically, the proposed scheme considers gathering backup channels in advance and using fuzzy logic for the selection of the best available backup channel.

Other studies have addressed the issue of user mobility in solving CR problems. For example, Sivasundarapandian et al. [27] present an approach based on a secondary user (SU) mobility model that considers SU movement and distribution. Dey and Saha propose a scheme that considers channel availability, interference, channel quality, and user distance to define the parameter MOS [28]. Al-Dulaimi et al. [29] propose a congestion control scheme, and Zhang et al. [30] propose an opportunistic programming scheme in the spectrum. However, none of these schemes solve the problem of effective handoff, nor do they consider a decision-making process that can help CR communication processes when there is GM.

The literature thus offers a wide range of proposals that consider mobility strategies. However, none of these proposals do not posit a decision-making process to determine the best communication channels, which results in ineffective handoff. In addition, the attributes that characterize a communication channel are not specified through the Delphi method [2]. To gain insights into the latency time of the proposed algorithm, other algorithms were included for comparison purposes, such as Dijkstra, AHP, FAHP, and ATDDiM.

Table 1 presents a summary that allows the comparison of common research methods, main features, limitations, and other characteristics of the algorithms found in the literature.

## 3. Decision-Making Algorithm with Geographic Mobility (DMAGM)

The proposed algorithm is based on the weighted sum shown in (1), which is part of a conventional technique for solving multi-objective optimization problems [30]. To find a better channel in a CR environment, with the efficiency and simplicity of using a linear combination of weights, some attributes such as the signal-to-interference plus noise ratio (SINR), the bandwidth (BW), the channel availability probability (AP), and the estimated channel time availability (ETA) are evaluated using the random way-point mobility model (RWPM). These criteria are considered for two types of services, i.e., real time (RT) and best effort (BE). In addition, the attributes are compared with those considered in [2] with a calculation of the weights to evaluate the channels that provide the best performance.

Equation (1) shows the objective function and attributes used for analyzing communication channels.

(1) $$Y=\sum_{j=1}^{n}w_{j}f_{j}\left(x\right),$$

where $w_{j}\;\in \;[0,\;1]$, and $f_{j}\left(x\right)$ is the *j*th objective function.

Considering that it is necessary to determine the channel with the best ‘fitness’/‘aptitude’, applying Equation (2), i.e.,

(2) $$C_{i}=w_{bw}^{ts}BW+w_{sinr}^{ts}SINR+w_{ap}^{ts}AP+w_{eta}^{ts}ETA,$$

where

*i*: id-number of one of the *N* channels to be compared
*ts*: type of service (RT or BE)
*BW*: normalized values of BW detected by the CR for channel *i*
*SINR*: normalized values of SINR detected by the CR for channel *i*
*AP*: normalized values of AP estimated by the CR for channel *i*
*ETA*: normalized values of ETA estimated by the CR for channel *i*
$w_{bw}^{ts}\;$: assigned weight to BW depending on the selected *ts*
$w_{sinr}^{ts}\;$: assigned weight to SINR depending on the selected *ts*
$w_{ap}^{ts}\;$: assigned weight to AP depending on the selected *ts*
$w_{eta}^{ts}\;$: assigned weight to ETA depending on the selected *ts*

**Figure 1:**
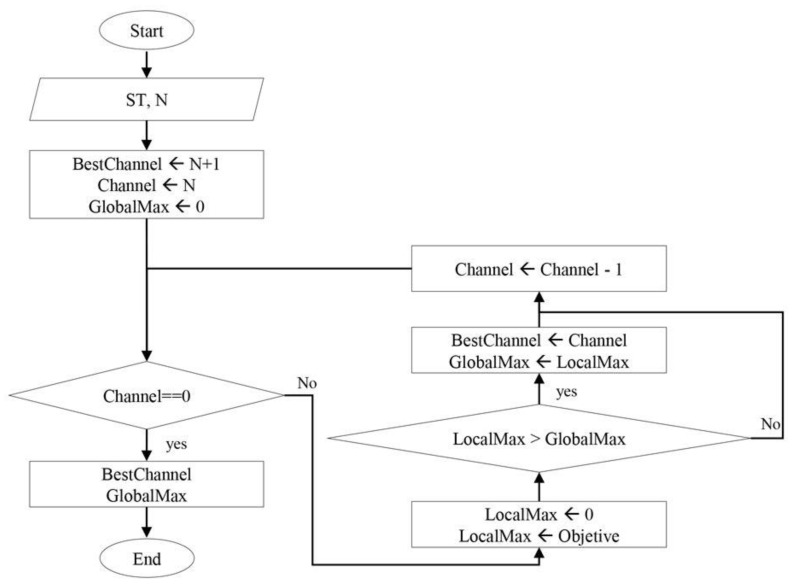
Proposed algorithm (DMAGM) diagram.

The values of BW, SINR, AP, and ETA, detected by the CRs, must be normalized in order to perform the weighted sum of multiple objectives. This sum is referred to as the ‘objective function’. All the objective function’s ‘aptitudes’ corresponding to the ‘N’ channels to be compared are thus obtained, computing a ‘global maximum’ among the set of channels and determining which channel has optimal communication characteristics. Figure 1 shows the flowchart of the DMAGM operation. Note that the input values are the ‘service type’ (ST) and the number of channels to be compared (N). The ‘type of service’ used consists of RT or BE.

Table 2 shows the selected values, which are values like [2] in order to compare performance. Initially, for the ‘best channel’s’ value to be optimized, a channel is placed outside the range of channels to be analyzed, and a channel within the range of channels is determined. If, at the end of the proposed algorithm, the value of the ‘best channel’ is outside the range of channels, the algorithm will not be able to determine a channel with the best ‘fitness’. The ‘global maximum’ value starts with a value of 0, which is the ‘fitness’ and represents that it will never have a channel analyzed, ensuring that the ‘global maximum’ value may be modified at least once. The ‘objective function’ for each channel is determined within the cycle shown in Figure 1, a value called the ‘local maximum’, which is compared with the value that demonstrates the ‘global maximum’. If the value is greater, then the ‘global maximum’ takes the value of the ‘local maximum’, and the ‘best channel’ assumes the value of the channel that is currently being analyzed. If the value is lower, the analysis of the next channel continues without modifying the values of the ‘best channel’ and the ‘global maximum’. The flowchart shows that this repetitive cycle continues until there are no more channel ‘fits’ to calculate, which is controlled by the ‘channel’ variable. Once the channel comparison cycle is complete, the output values with the ‘best channel’ and the ‘global maximum’ determined by the DMAGM are displayed.

At this stage, a feedback mechanism is implemented in order to improve the accuracy with which a channel with the best communication characteristics can be determined. This procedure allows consulting the history of previous results. The process to improve the precision in selecting a channel is described below.

### Feedback-Decision-Making Algorithm with Geographic Mobility (FDMAGM)

**Figure 2:**
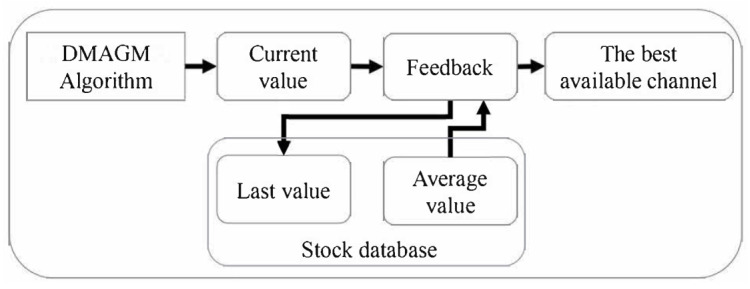
The feedback-decision-making algorithm scheme (FDMAGM).

The feedback-decision-making algorithm with geographic mobility (FDMAGM) considers increasing the accuracy in selecting the best channel in a similar way to [12]. The feedback process obtains information from previous evaluations of the channels. The value allocated to each channel corresponds to a relationship between current information and past evaluations. As can be seen in Figure 2, the FDMAGM is based on the original DMAGM but includes a feedback process.

A process to determine the new ‘best channel’ is carried out that considers the current value, the last generated value, and the average value of a certain time interval. The final value for each channel is determined using (3).

(3) $${SF}_{i}=\alpha •SA+\beta •SPa+\left(1-\alpha -\beta \right)•SPr,$$

where

*i*: id-number of one of the N channels to be compared
*SF*: final value
*SA*: current value
*SPa*: previous value
*SPr*: mean value
α, β ∈ [0, 1]

Once the final value is obtained for each channel, a comparison is made with the decision-making process outlined above, thus determining the best channel among the set of channels analyzed. The values of α and β are obtained in a similar way as in [12] by performing an experimental auto-regressive analysis with different combinations of α and β for a set of predetermined data. The values of α and β were considered so that the precision in the selection of the best channel was higher. These values are α = 0.60 and β = 0.35, with an experimental precision of 87%. Both algorithms were analyzed with the two types of services: RT and BE, as well as with normalized weights.

## 4. Tests and Result Analysis

**Figure 3:**
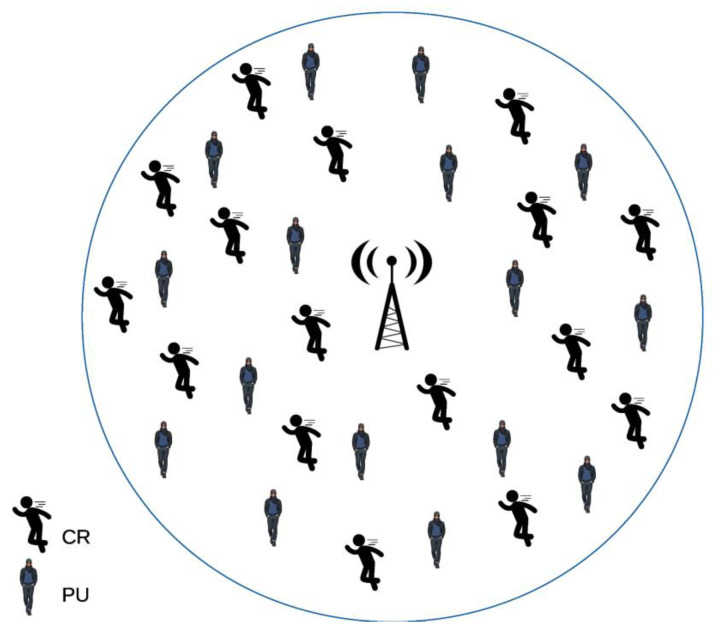
Geographic mobility scenario of a CRN with one BS.

The algorithm proposed in this study for decision-making (DMAGM) has the characteristic of improving the response time (latency). The test scenario for the evaluation and validation of the DMAGM and the FDMAGM was configured with the services of RT and BE using Network Simulator 3 (NS-3). Table 3 shows the network characteristics in which it is considered that a base station (BS) with cognitive radio characteristics receives all the information detected by the CRs, i.e., the main characteristics and attributes of the channels observed and selected during previous analyses, which are: BW, SINR, AP, and ETA found within the range of channels that can be detected by the system. Initially, as shown in Figure 3, the behavior of the proposed algorithms, the DMAGM and the FDMAGM, is considered with and without a feedback mechanism, in a scenario with GM in which there is interaction of cognitive radios (CRs) and primary users (PUs). Subsequently, the latency in relation to the DMAGM is obtained and compared with the Dijkstra [10], AHP [11], FAHP [12], and ATDDiM [13] algorithms. Finally, the results obtained for the DMAGM are shown and compared with the FDMAGM (applying the feedback mechanism).

### 4.1. Simulation Scenario with Geographic Mobility

Figure 3 shows an approximation of the BS’s geographical positions, primary users (PUs), and cognitive radios (CRs). In addition, considering that the decision-making algorithm is developed by the BS, it therefore assesses the selection of the best communication channel and establishes which channels can be occupied by the CRs. The mobility pattern followed by the CRs and PUs is defined by the random way-point mobility model (RWPM), and mobility simulation time has a duration of 3600 s. Consequently, the behavior of CRs and PUs is modeled and simulated using 124 randomly distributed nodes on a circular surface with a radius ranging from 2167 m to 4334 m, in which each node is allocated with a transmission channel.

In this evaluation, three GM scenarios with different occupancy percentages were generated for each channel, considering values between 25% and 75% in 25% intervals. For each of these three scenarios, three variations in PU mobility were proposed by modifying the PU mobility radius in relation to the BS coverage radius. That said, the following distances were considered:

- Same as the BS coverage radius, i.e., 2167 m
- 50% greater than the BS coverage radius, i.e., 3250.5 m
- 100% greater than the BS coverage radius, i.e., 4334 m

The simulation results yielded the average time that a channel is available. For the proposed scenarios, a simulation time of approximately 1.48 s corresponding to 2400 samples per scenario was tested. Table 4 shows the results obtained from both algorithms, with and without the feedback mechanism. Note that in the selection of the best channel, there is a similarity above 75% for the different scenarios. Therefore, algorithm application and implementation will depend on the scenario in which they are tested.

### 4.2. Comparison of the Decision-Making Algorithm with Geographic Mobility

In addition, for the purpose of comparison with existing algorithms, similar attributes as the ones presented in [2] were considered. In the first part of the tests, the algorithms’ computational latency was obtained: Dijkstra [10], AHP [11], FAHP [12], ATDDiM [13], and the DMAGM using pseudo-randomly generated channels with a triangular distribution with normalized values from 3 to 38 for the ETA, 33 to 98 for the AP, 0 to 10 for the SINR, and a single value of 200 for the BW. Triangular distribution is widely used as a general approximation of any central tendency distribution (average value) and when its measure of dispersion is loosely known.

**Figure 4:**
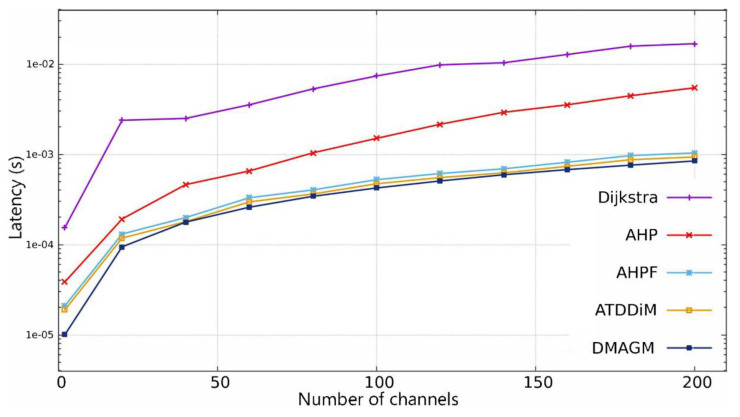
Latency comparison among several decision-making algorithms considering 200 channels.

Figure 4 shows that the DMAGM has a low latency with respect to the other algorithms for 200 channels, which reveals that the time to indicate the best channel is the lowest with respect to the other algorithms.

It should be noted that each of the points shown in Figure 4 was obtained with an average of 100 repetitions of algorithms using different inputs. Then, the latency for each set of channels to be compared is calculated.

**Figure 5:**
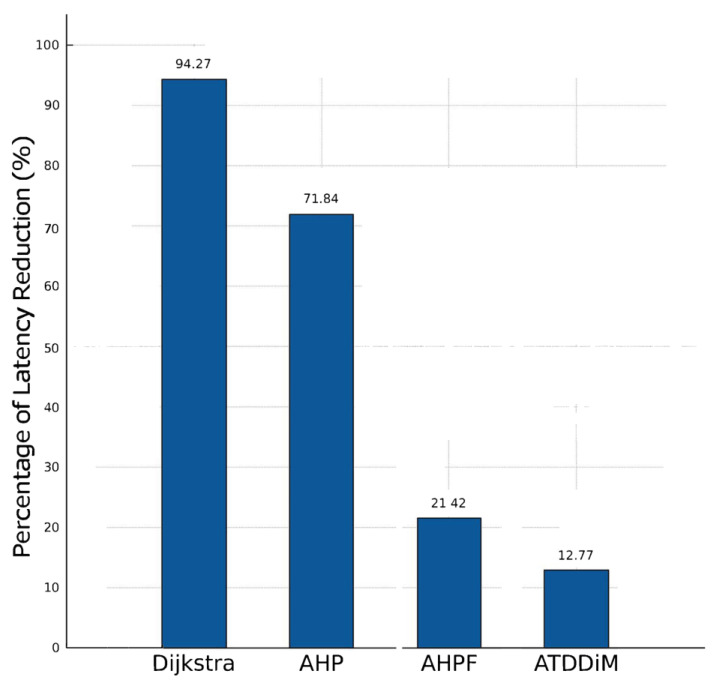
Percentage of latency reduction comparison of DMAGM with respect to reference algorithms.

Figure 5 shows the percentage of time of the proposed algorithm DMAGM with respect to the other algorithms evaluated. Note that decision-making latency for the DMAGM is significantly reduced by 12.77% compared with ATDDiM, which is the second most effective algorithm and followed by FAHP with 21.42%, AHP with 71.84%, and Dijkstra with 94.27% in terms of latency reduction.

### 4.3. Analysis of the Feedback-Decision-Making Algorithm with Geographic Mobility

According to the results obtained above, and in order to validate the performance of the proposed algorithm, a comparison between the FDMAGM and the DMAGM was developed, i.e., with and without considering the feedback mechanism.

**Figure 6:**
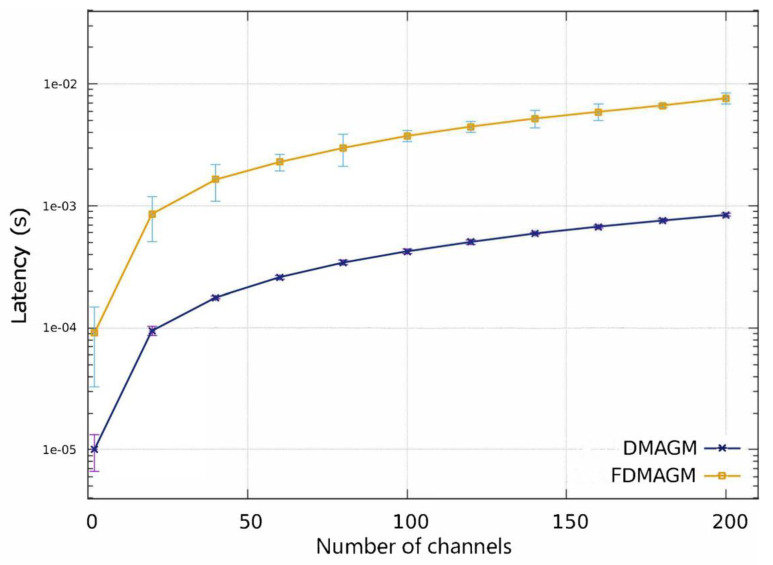
Latency comparison between FDMAGM and DMAGM (with and without a feedback mechanism) with 95% confidence intervals for each number of channels tested.

Figure 6 shows the number of channels and their respective latency, which initially considers 2 channels, then 20 channels, and subsequently increases by 20 channels until reaching 200 channels.

Figure 6 also shows the confidence intervals with a level of 95% for 200 analyzed channels. Channels were randomly generated using a triangular distribution with normalized values from 3 to 38 for the ETA, 33 to 98 for the AP, 0 to 10 for the SINR, and a single value of 200 for the BW. Subsequently, to obtain the confidence interval and determine which channel is the best with respect to the proposed algorithm with and without a feedback process, the averages of 100 repetitions were computed for each of the proposed algorithms with different inputs.

In addition, Figure 6 shows that the latency of the proposed algorithm with feedback (FDMAGM), on average, increases nine times with respect to the latency of the proposed algorithm without feedback (DMAGM). This is due to the mechanism used in the feedback to check the previous state of the current channel. Consequently, according to the results, it can be suggested that the FDMAGM (with a feedback mechanism) is useful in cases in which the processing time is not an important factor when determining a channel with the best communication characteristics and with significant results in the new determination of a better communication channel.

Table 5 shows the results of the compared channels, which indicates that there is a 95% confidence level in the latency it takes to select the communication channel.

## 5. Conclusions

This work provides a novel proposal that considers multivariable decision-making for cognitive radio (CR) with geographic mobility (GM). The consulted literature shows scarce alternatives for channel allocation times when assessing the GM for the cognitive radio network (CRN), which requires in-depth analysis.

It is very helpful to use GM information in a CRN for several reasons such as reducing the number of secondary users that are connected to the network and where the quality of service can be affected. The literature reveals that decision-making is one of the main problems presented by CRNs since it is a fundamental operation for spectrum allocation, as well as spectrum sensing, spectral mobility, and sharing and cooperation. The decision-making algorithm with geographic mobility (DMAGM) is crucial for improving the channel selection and spectrum mobility.

Simulations demonstrate that the DMAGM reduces the processing time between 12% and 94% compared with other algorithms. In addition, the analysis considers geographic mobility (GM), which is, to the best of the authors’ knowledge, not found in the literature for CRNs. In addition to the DMAGM, a modification to the algorithm was developed and tested using a feedback mechanism, named in this research as the feedback-decision-making algorithm with geographic mobility (FDMAGM). The FDMAGM was designed with the purpose of not only improving latency in decision-making but also to explore other options to improve the quality of the selection for the communication channel. The parameters considered for the analysis are based on a customized Delphi method, which are the bandwidth (BW), the signal-to-interference plus noise ratio (SINR), the channel availability probability (AP), and the estimated channel time availability (ETA).

Additionally, the history of previous results in the FDMAGM regarding the best selected communication channel is considered. As shown in the results, the processing time of the FDMAGM increased on average compared with the DMAGM. However, the FDMAGM can efficiently be used in the proposed scenarios without significant changes in the network. The feedback system has the advantage of being able to continually adjust and adapt based on the feedback received. In addition, the feedback version helps to identify and correct problems, which can be beneficial in situations where the quality of communication is critical. Despite the fact that the FDMAGM may take longer than the DMAGM to calculate the best communication channel, constant feedback improves efficiency and effectiveness over time. Both the DMAGM and the FDMAGM improve performance in practical scenarios, the former in terms of latency and the latter in terms of accuracy and stability.

Both the DMAGM and the FDMAGM have proven to be useful depending on the applications tested, such as best effort (BE) and real time (RT), and the different environments shown varying the radius of operation. This demonstrates that the match rate of these algorithms is similar in the worst-case scenario, about 75% on the selection of the best communication channel considering 2400 samples per scenario with the random presence of primary users (PUs).

## Figures and Tables

**Table 1:** Main characteristics of different decision-making algorithms for cognitive radio, where *n* represents the number of nodes and *m* represents the number of channels. The acronym NC means not considered, and NA means not available.

Algorithm	Geographic Mobility (GM)	Computational Complexity	Min./Average/Max. Decision-Making Latency (ms)	Main Features	Limitations
Dijkstra [10]	NC	$O(n^{2})$	0.2/8.6/20	This approach can find the optimal path between two nodes in a graph. This approach can be used to make decisions in a variety of scenarios. This approach can be scaled to large networks.	This approach is sensitive to the accuracy of the data on the structure of the graph.
AHP [11]	NC	$O(n^{2})$	0.04/2.2/6	Identifies the most cost-effective and latency-optimal routes between two points. Simplifies the route selection process for delivery services. Allows the user to specify the weights of the decision factors.	It depends on the accuracy of the specified weights by the user. It might be unreliable in networks with changing conditions, like mobility scenarios.
FAHP [12]	NC	$O(n^{2})$	0.02/0.47/1	Improves the performance of spectral handoff by more efficiently selecting channels. It can be adaptable to different cognitive radio network scenarios.	It requires a large amount of data about the cognitive radio network. It is more complex than traditional spectral handoff algorithms. It depends on the accuracy of the specified weights by the user.
ATDDiM [13]	NC	O(nlog(n))	0.02/0.46/1	Enhances route planning performance by reducing computation time compared with Dijkstra. Reduced computation complexity. The improved algorithm implements a configurable set of numerical parameters.	It requires more preprocessing power than the original Dijkstra’s algorithm.
MAS [14]	NC	$O(n^{2})$	NA	Spectrum efficiency using a chaotic channel selection algorithm. The scheme is robust to changes in the radio environment. Easy to implement.	The mobility scheme requires data on the structure of the cognitive radio network. The mobility scheme is sensitive to the accuracy of the data on the structure of the cognitive radio network.
Cluster- based MAC protocol [15]	NC	O(nlog(n)+m)	NA	Energy efficiency. Robustness because of the mobile clustering scheme. Easy to implement.	The protocol does not use a mobility prediction model to estimate the future location of nodes. The protocol does not take into account the impact of mobility on network performance.
CSA [21]	NC	O(nlog(n)+m)	NA	Improves the performance of cognitive radio networks in terms of transmission success rate and signal quality. The algorithm is robust to interference.	The model does not consider user mobility to estimate the probability of interference-free channels at a given time. The model does not consider interference from other users to estimate the probability of occupied channels at a given time.
SHMA [23]	NC	O(nlog(n)+m)	NA	The proposed algorithm improves spectrum efficiency by selecting the best channel for each mobile user. The proposed algorithm improves QoS by considering the reception gain, interference, and other factors when selecting channels. The proposed algorithm is adaptive to mobility by considering the mobility of the user when selecting channels.	The algorithm assumes that the user’s spectral mobility is known. The algorithm does not consider the impact of spectral mobility on interference. The algorithm does not consider the impact of spectral mobility on network congestion.
MOS [28]	NC	O(mlog(n)+n))	NA	The scheme is robust to interference because it considers the interference from primary users when making spectrum handoff switching decisions. The scheme is adaptive to mobility because it considers the mobility of secondary users when making spectrum handoff switching decisions.	The scheme assumes that primary users behave predictably. They are the only considered parameter for spectrum handoff without geographic mobility.

**Table 2:** Weights of attributes according to the type of service used.

Criteria	RT	BE
*BW*	0.1471	0.2921
*SINR*	0.1970	0.3949
*AP*	0.3593	0.1607
*ETA*	0.2966	0.1523

**Table 3:** Parameters used in the simulation scenario with NS-3.

Parameters	Value
Frequency band	824–849 [MHz]
Communication system	Mobile
Communication technology	GSM
Number of channels	124
BW per channel	200 [kHz]
Power Tx BS	30 [dBm]
BS coverage area	2167 [m]
BS height	25 [m]
Mobile user Rx power	−80 [dBm]
Mobile user height	1 [m]

**Table 4:** Match rate between FDMAGM and DMAGM, with and without the feedback mechanism, for the selection of the communication channel.

Characteristics of the GM Scenario		Number of Matches over 2400 Samples	Match Rate
Mobility Radius	CPU Occupancy		
2167 m	25%	1819	75.79%
	50%	2062	85.91%
	75%	2128	88.66%
3250.5 m	25%	1839	76.62%
	50%	2090	87.08%
	75%	2162	90.08%
4334 m	25%	1821	75.87%
	50%	2075	86.45%
	75%	2190	91.25%

**Table 5:** Average latency values for FDMAGM and DMAGM (with and without a feedback mechanism) with a confidence interval of 95% in the selection of the communication channel.

Algorithm	Analyzed Channels	$\mathrm{Average}\;\mathrm{Latency}\;\mathrm{Value}\;\bar{x}$ (s)	Confidence Interval (s)	
			$\bar{x}-1.96\cdot \frac{\sigma }{\sqrt{n}}$	$\bar{x}+1.96\cdot \frac{\sigma }{\sqrt{n}}$
DMAGM	2	1.0018 × 10^−5^	6.6341 × 10^−6^	1.3402 × 10^−5^
	20	9.4471 × 10^−5^	8.6513 × 10^−5^	1.0243 × 10^−4^
	40	1.7717 × 10^−4^	1.7216 × 10^−4^	1.8217 × 10^−4^
	60	2.5918 × 10^−4^	2.5102 × 10^−4^	2.6733 × 10^−4^
	80	3.4220 × 10^−4^	3.2805 × 10^−4^	3.5634 × 10^−4^
	100	4.2337 × 10^−4^	4.0347 × 10^−4^	4.4327 × 10^−4^
	120	5.0618 × 10^−4^	4.8179 × 10^−4^	5.3058 × 10^−4^
	140	5.9363 × 10^−4^	5.8207 × 10^−4^	6.0519 × 10^−4^
	160	6.7631 × 10^−4^	6.6335 × 10^−4^	6.8927 × 10^−4^
	180	7.5883 × 10^−4^	7.3926 × 10^−4^	7.7841 × 10^−4^
	200	8.4375 × 10^−4^	8.2255 × 10^−4^	8.6496 × 10^−4^
FDMAGM	2	9.0613 × 10^−5^	3.2976 × 10^−5^	1.4825 × 10^−4^
	20	8.5449 × 10^−4^	5.1269 × 10^−4^	1.1963 × 10^−3^
	40	1.6415 × 10^−3^	1.0943 × 10^−3^	2.1886 × 10^−3^
	60	2.2854 × 10^−3^	1.9420 × 10^−3^	2.6288 × 10^−3^
	80	2.9847 × 10^−3^	2.0981 × 10^−3^	3.8714 × 10^−3^
	100	3.7482 × 10^−3^	3.3557 × 10^−3^	4.1408 × 10^−3^
	120	4.4709 × 10^−3^	3.9891 × 10^−3^	4.9526 × 10^−3^
	140	5.2138 × 10^−3^	4.3504 × 10^−3^	6.0772 × 10^−3^
	160	5.9145 × 10^−3^	4.9912 × 10^−3^	6.8378 × 10^−3^
	180	6.6679 × 10^−3^	6.4288 × 10^−3^	6.9069 × 10^−3^
	200	7.6456 × 10^−3^	6.8606 × 10^−3^	8.4306 × 10^−3^

## Data Availability

Data are contained within the article.

## Abbreviations

CR	Cognitive Radio
CRN	Cognitive Radio Network
GM	Geographic Mobility
PU	Primary User
SU	Secondary User
BS	Base Station
BW	Bandwidth
SINR	Signal-to-Interference plus Noise Ratio
AP	Channel Availability Probability
ETA	Estimated Channel Time Availability
DMAGM	Decision-Making Algorithm with Geographic Mobility
FDMAGM	Feedback-Decision-Making Algorithm with Geographic Mobility
ATDDiM	Modified Dijkstra Decision-Making Algorithm
FAHP	Fuzzy Analytic Hierarchy Process
AHP	Analytic Hierarchy Process
RWPM	Random Way-Point Mobility Model
RT	Real Time
BE	Best Effort
GSM	Global System for Mobile Communications
NS-3	Network Simulator-3
